# Cost of hospital care for the older adults according to their level of frailty. A cohort study in the Lazio region, Italy

**DOI:** 10.1371/journal.pone.0217829

**Published:** 2019-06-11

**Authors:** Giuseppe Liotta, Francesco Gilardi, Stefano Orlando, Gennaro Rocco, Maria Grazia Proietti, Federica Asta, Manuela De Sario, Paola Michelozzi, Sandro Mancinelli, Leonardo Palombi, Maria Cristina Marazzi, Paola Scarcella

**Affiliations:** 1 University of Rome Tor Vergata, Department of Biomedicine and Prevention, Rome, Italy; 2 IPASVI Centre of Excellence, Rome, Italy; 3 OPI, Rome, Italy; 4 Regione Lazio Department of Epidemiology, Rome, Italy; 5 L.U.M.S.A. University, Rome, Italy; university of campus biomedico, ITALY

## Abstract

**Background:**

The increasing burden of chronic diseases associated with the ageing of the European population constitutes one of the main challenges for the welfare systems in developed western countries, especially through its impact on the use of hospital services and the cost of care. This study aims at evaluating the cost of hospital care for older adults living in the Lazio Region, Italy, according to their level of frailty.

**Methods:**

Since 2014 a longitudinal randomized cohort study has been carried out on a sample consisting of 1280 older adults aged over 64 years resident in the Lazio region (Italy), with their being evaluated for multidimensional frailty. Accesses to Hospital Services (acute care and Day Hospital care admissions and Emergency Room accesses) during the first year after enrolment, as well as the related costs have been recorded through a regional database. Costs have been stratified on the basis of the state of frailty.

**Results:**

The analysis of hospital services and costs highlights the role played by pre-frail individuals who generated 49.3% of the hospital care cumulative costs. Hospital Admission (HA) costs arising from robust and pre-frail subjects are 70% of the total HA costs. Pre-frail individuals also showed the highest average HA cost per person/year (7062.89 Euros). The main determinant of the highest HA costs was given by the number of HAs during the follow-up (multivariate linear regression, ß coefficient = 0.319; p<0.001), which was higher among pre-frail individuals than in any other group of patients.

**Conclusions:**

Pre-frail individuals generated the highest cost for hospital care in a sample of representative subjects living in an Italian Region with a low rate of community care services, as is the case in the Lazio region. Assessment of the multidimensional frailty of older adults permits a better definition of the important target of the pre-frail population as the main category within which interventions to prevent or mitigate frailty should be carried out.

## Introduction

The increasing burden of chronic diseases associated with the ageing of the European population constitutes one of the main challenges for the welfare systems in developed western countries [1]. The demographic and epidemiological transitions translate into an increase of care demand that could threaten the sustainability of health systems. Although the current older generations appear healthier and more active than previous ones, the growing number of older adults is leading to a net increase in the burden of disease due to chronic pathologies [2–4]. Economic analyses worldwide recognize the central role of this demographic phenomenon and of its impact on our social and health systems [5], as well as the need for successful strategies aimed at improving both the sustainability of health and welfare systems and people’s quality of life [6,7].

In Italy the cost for hospital care accounts for 45.5% of total health expenditure, the second highest cost in percentage terms among the European Union (EU) countries [8]. Acute care is the main driver of hospital health expenditure, since it accounts for more than 90% of hospital care costs, with about 55% of this expenditure being generated by hospitalizations of patients over 64 years of age [9–11]. Interestingly, while total hospital health expenditure has decreased over the years, that generated by older adults is increasing both in absolute numbers and as a percentage [9–11].

However, the causes of the increase in cost of health care arise from more specific factors than from population ageing *per se*: these factors include the increased cost of new health technologies, Socio-Economic Status (SES) and Time-To-Death (TTD), which is a proxy for the increase of frequency and/or severity of multimorbidity [12–16]. Defining the main determinants of hospital care costs generated by older adults seems to be crucial for planning interventions aimed at increasing the sustainability of health systems.

The major determinants of hospitalization rate among the older adults have often been discussed by researchers who, most of the time, have focused on specific diseases. When the analysis mainly referred to older adults, the discussion was essentially centered on multimorbidity, since many older patients are affected by more than one disease concurrently [17,18]. Analysis of the relationship between multimorbidity and Use of Hospital Services (UHSs) also shows an association between multimorbidity classes and the highest UHS and socio-economic factors [17]. Therefore, the impact of non-clinical factors on UHS should be taken into account as a major determinant. This is the case if UHS stratification risk is based on an assessment of bio-psycho-social frailty that can provide a strong UHS determinant either as frailty *per se* or as components of frailty, such as social isolation, disability, psychological and psycho-physical impairment [19–22]. Nonetheless, studies of the cost associated with the state of frailty are mainly based on the bio-medical definition of frailty, such as the best known one proposed by Fried [23–29]. The use of the bio-psycho-social definition of frailty, that includes both bio-medical and socio-economic factors [30], led to the conclusion that long-term care costs are mainly driven by the state of frailty [31].

The aim of this paper is to highlight the impact of bio-psycho-social frailty on hospital care costs.

## Methods

Since the beginning of 2014, a longitudinal cohort study has been carried out based on a sample consisting of 1280 older adults (age>64, resident in the Lazio region (Italy)). The sample was selected and enrolled during 2014 by block randomization in order to be fully representative of the population aged over 64 years in the Lazio region [32]. The randomization was performed on the basis of:

1. Regional municipality, refering to one Local Health (LHA), randomized on the basis of population, geography, dependency index and socio-economic status
2. General Practitioners (GPs) to be involved in the study, identified through randomization of the LHA list
3. GP‘s lists of patients over 64 years. A maximum of 25 patients per GP were chosen through randomization of the GP’s list of patients over 64 years of age.

The patients over 64 not residing at home were excluded from the selection process.

After being selected, study candidates were contacted by phone by their GP in order to give initial consent to the interview that was administered in the GP’s outpatient facility or at the patient’s home if the patient was unable to travel.

Based on the number of citizens over age 64 years residing in the Lazio region, for a precision of 3% with a 95% confidence limit, taking into account a refusal rate of 10%, we estimated 1300 citizens were required to power the study.

The enrolled subjects were screened for frailty through the administration of a validated multidimensional questionnaire (the Functional Geriatric Evaluation–FGE) [32]. Based on the questionnaire score, the interviewed subjects were classified into four categories (robust, who scored more than 70 points, pre-frail between 50 and 69, frail between 11 and 49 and very frail less than 11), according to the questionnaire score validation [33]. The questionnaire also includes a list of diseases to be filled in in collaboration with General Practitioners (GP). During the follow-up, accesses to the Hospital Services (HS) (acute care and Day Hospital care admissions and Emergency Room–ER—accesses) and their related costs have been obtained from the regional databases (Hospital Information System (HIS), Healthcare Emergency Information System (HEIS) of the Lazio Region).

The cost analysis was carried out based on an evaluation by the service provider and the third payer, in this case the Lazio regional administrative body. Costs incurred by patients or their families, or costs incurred by others beyond the health system were not considered.

The regional database on the use of HS was the source of information used for the cost analysis. The cost of hospital admissions is based on Diseases Related Group (DRG) classification and represents the real cost determined by the regional administration for each hospital admission. According to the most recent legislation of the Lazio Region, an average cost has been used for the ER accesses [34]. Costs have been stratified on the basis of the state of frailty.

The study was approved by the independent Ethics Committee of the University of Rome Tor Vergata (Protocol Nr 95/15, dated 25.07.2015; 0016145/2015). A written informative consent form was administered to each subject involved in the study.

A descriptive analysis was carried out on UHS rates by level of frailty. The Kruskal-Wallis test was performed in order to compare average costs and UHSs among the frailty categories. The Pearson chi-square test was used to assess differences between categorical variables. T-Test and ANOVA were used to select the variables to be included in the multivariable linear regression model on the UHS’s cost determinants. A two-sided p-value <0.05 was considered statistically significant. The Multiple Correspondence Analysis technique has been performed to characterize the patients according to the association of different diseases as per the diagnosis reported by the GPs. Missing data were always less than 5% of the sample and were excluded from the analyses, assuming that their distribution does not affect the validity of the analyses. SPSS statistical Package 22.0 was used to perform the statistical analysis.

## Results

The sample consists of 591 (46.2%) males and 689 females (53.8%), the mean age is 76.3 years (SD±7.1).

Following the administration of the questionnaire, the subjects were divided into 4 categories: robust (549 interviewed, 42.8%), pre-frail (459, 35.9%), frail (175, 13.7%) and very frail (97, 7.6%) based on the questionnaire score [33]. Some of the main baseline characteristics of the sample are reported in Table 1.

**Table 1:** Baseline general characteristics of the study population*.

	Robust	Pre-frail	Frail	Very frail	Anova p-value
**Age**:mean (SD)	73.35 (±6.00)	76.95 (±6.33)	79.68 (±7.51)	83.72 (±6.94)	<0.001
Multimorbidity**: mean (SD)	2.8 (±1.9)	3.5 (±2.2)	4.6 (±2.4)	5.4 (±2.7)	<0.001
					**Chi-square p-value**
**Gender** (female %)	45.6	55.0	69.1	67.0	<0.001
**Education** (High School Degree %)	54.1	44.8	29.3	28.9	<0.001
**Living arrangements** (Living alone %)	7.7	32.8	32.6	15.5	<0.001
**Disability** (%)***	93.4	72.8	19.4	1.0	<0.001

*These parameters do not contribute to the final frailty score that is calculated through the answers to the questionnaire, except for disability, which is calculated by assessing ADL in a different way

**The number of diseases as reported by the GPs intheir clinical files

***No impairment in performing ADL/IADL

Overall, 386 out of 1280 interviewed subjects used at least one HS (30.2%) during the first year of follow-up: a clear trend associated to the state of frailty can be observed for the percentage of subjects accessing HSs (Table 2). The three categories of frail and pre-frail individuals showed rates higher than for robust individuals (p<0.004). The average number of HSs used by the interviewed subjects who accessed hospital care at least once during the first year of follow-up was 2.06 (SD±1.52); frail people showed the highest UHS mean rate (2.40 per person, SD±1.61), higher than very frail (2.25, SD±1.25) and pre-frail (2.14, SD±1.77) (Table 2).

**Table 2:** Hospital care services by level of frailty.

Frailty	Number of persons	AccessedHSs	%	UHSs (Nr)	UHSs (mean)	SD	Kruskal-Wallis test p-value
Robust	549	131	25.7	231	1.76	1.17	0.006
Pre-frail	459	140	30.5	300	2.14	1.77	
Frail	175	60	34.3	144	2.40	1.61	
Very frail	97	36	37.1	81	2.25	1.25	
Total	1280	386	30.2	756	2.06	1.52	

**Fig 1:**
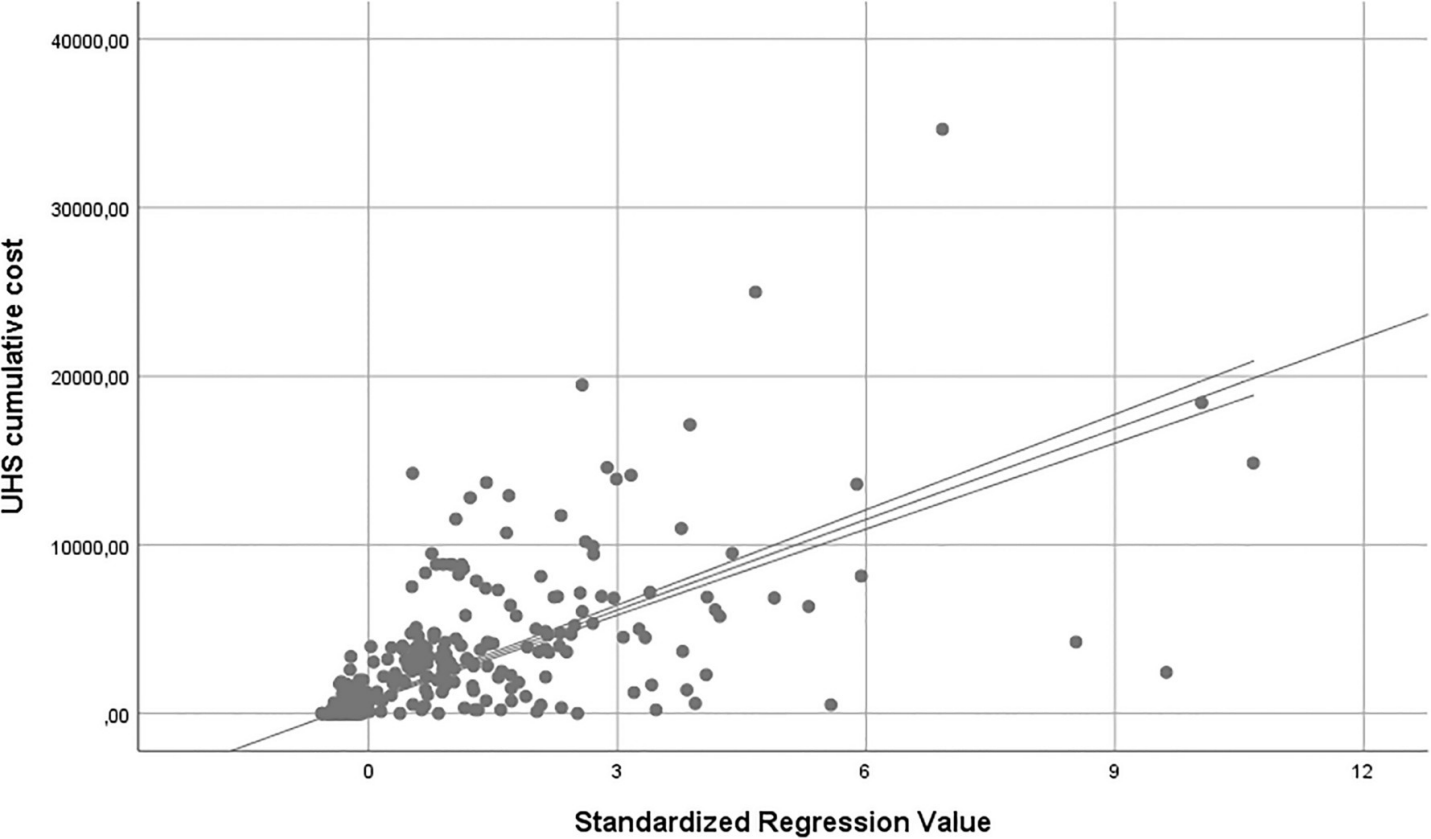
Determinants of UHSs cost. (multivariable linear regression; outcome variable: cumulative UHSs costs; R^2^ = 0.526).

The mean cost for UHSs per person was 5186.94, 8274.44, 5662.40, and 6308.96 Euros for robust, pre-frail, frail and very frail individuals, respectively (p = 0.006). The variables to be included in the multivariable model of predictors of higher UHS expenditure were the ones associated with higher mean UHS cost at the univariate comparison (T-TEST/ANOVA, p<0.05), such as the presence of Cardiopathy, Chronic Obstructive Pulmonary Disease (COPD), Cancer, Parkinson Disease, Cognitive decline and Nephropathy. Some variables have been included in the multivariable model independently from the generated UHS cost because of their relevance at population level: gender, age, disability (any impairment on ADLs/IADLs), number of diseases that affected the subjects as retrieved by the GPs’ clinical files (multimorbidity), Frailty Score. The multivariable linear regression showed a statistically significant association between UHS total costs and increasing UHS rate per person/year and length of hospital stay, excluding all the diseases from the model (Table 3; Fig 1).

**Table 3:** Multivariable linear regression. Outcome variable: total UHSs’ costs (386 individuals, adjusted R^2^).

Model	β	p-value
Hospital Admission rate per person/year	0.474	<0.001
Length of stay (cumulative per person)	0.142	<0.001

Adjusted for Age, Gender, Frailty score, Disability (any impairment in ADL/IADL) and Multimorbidity that did not reach a statistical significance

Because the UHS cost was not normally distributed, however, we also performed a linear regression analysis using the logarithm of the UHS cost, this being approximately normally distributed (S1 Table). This only resulted in the additional inclusion of Cancer in the pool of predictors of higher UHS cost, with a marginal contribution to the model’s standardized R^2^ (S2 Table).

The analysis of UHS events and costs highlights the role played by pre-frail individuals in generating hospital care costs: in fact, pre-frail patients generated 49.3% of the cumulative cost and 50.2% of the costs due to HA. HA costs are the source of close to 90% of the total hospital services costs generated by the cohort, with 70% of these being due to the admissions of robust and pre-frail subjects (p = 0.076)–which are probably partially preventable. Pre-frail individuals also showed the highest average HA cost per person (7,062.89 Euros) (Table 4).

**Table 4:** Use and cost of hospital services according to the level of frailty (persons who accessed hospital services at least once during the first year of follow-up).

**Hospital admission (HA)**							
Frailty	Number of Persons Admitted (NPA)	Number of HAs	% HAs	HA per person (NHAs/ NPA)	Total Cost (TC) (Euros)	% TC	A1—Cost per person (TC/NPA)
Robust	47	55	25.2	1.17	179 408.00	20.6	3817.19
Pre-frail	62	96	44.0	1.55	437 899.00	50.2	7062.89
Frail	31	44	20.2	1.42	143 960.12	16.5	4643.88
Very frail	18	23	10.6	1.28	110 525.00	12.7	6140.28
Total	158	218	100.0	1.39	871 792.12	100.0	5517.67
p-value							0.076
**Emergency Room visits (ERv)**							
Frailty	Number of Persons who Accessed the ER(NPAER)	Number of ERv	% ERv	ERv per person (ERv/ NPAER)	TotalCost (TC)	% TC	A2- Cost per person (TC/ NPAER)
Robust	101	153	31.7	1.51	16 019.10	31.6	158.60
Pre-frail	111	179	37.1	1.61	18 741.30	37.1	168.84
Frail	53	93	19.2	1.75	9 737.10	19.3	183.72
Very frail	36	58	12.0	1.61	6 072.60	12.0	168.68
Total	301	483	100.0	1.60	50 570.10	100.0	168.00
p-value							0.013
**Day Hospital admissions (DHa)**							
Frailty	Number of Persons Admitted (NPA)	Number of DHa (NDHa)	% DHa	HA per person (NDHa/ NPA)	Total Cost (TC)	% TC	A3- Cost per person (TC/NPA)
Robust	19	25	45.4	1.14	23 012.00	46.8	1211.15
Pre-frail	21	23	41.8	1.32	21 897.00	44.5	1042.71
Frail	5	7	12.7	1.40	4 274.00	8.7	854,80
Very frail	0	0	0.0	0.00	-	-	-
Total	45	55	100.0	1. 22	49 183.00	100.0	1092.96
p-value							0.055
**Cumulative Hospital Services (HSs)**							
Frailty	Number of Persons	Number of HSs	% of HSs	HSs/ person	Total Cost (TC)	% TC	A1+A2+A3Cost/ person
Robust	131	231	30.6	1,76	218 439.10	22.5	5186.94
Pre-frail	140	300	39.7	2.14	478 537.30	49.3	8274.44
Frail	60	144	19.0	2.40	157 971.30	16.3	5662.40
Very frail	36	81	10.7	2.25	116 597.60	12.0	6308.96
Total	367	756	30.8	2.06	971 545.30	100.0	6778.63
p-value							0.006

The main determinant of the highest Has’ costs is the number of HAs during follow-up (as determined by multivariate linear regression adjusted for FSS, age, gender, and multimorbidity; ß coefficient = 0.319; p<0.001), which was higher among pre-frail individuals than in any others. The cumulative length of stay was not associated with higher costs, while a diagnosis of COPD in the patient’s clinical history (even if it was not necessarily the cause of the admission) was weakly associated with increased HA costs (ß coefficient = 0.075; p<0.001). Among 40 subjects who experienced multiple hospital admissions, 18 (45.0%) pre-frail generated 50 out of 100 admissions (50.0%) and 19.0% of the total costs for HAs. Overall, persons who experienced multiple admissions accounted for 3.1% of all the interviewees and generated close to 36.0% of the total HA costs (Table 5).

**Table 5:** Hospital admission (HA) cost per single or multiple hospital admissions, according to the level of frailty.

	State of frailty	Number of persons	Number of HAs	Mean HA per person	Mean cost per person	SD	Total Cost	% of total cost
One hospital admission	Robust	39	39	1	3715.46	3395.56	144 903.00	26.0
	Pre-frail	44	44	1	5754.68	5687.83	253 206.00	45.4
One hospital admission	Robust	39	39	1	3715.46	3395.56	144 903.00	26.0
	Pre-frail	44	44	1	5754.68	5687.83	253 206.00	45.4
	Frail	21	21	1	4096.10	2974.44	86 018.20	15.4
	Very frail	14	14	1	5245,29	4560.75	73 434.00	13.2
	**Total**	**118**	**118**	**1**	**4725.09**	**5153.00**	**557 561.20**	**100.0**
Multiple hospital admissions	Robust	8	16	2.00	4313.12	2070.75	34 504.96	11.0
	Pre-frail	18	52	2.89	10260.72	7837.76	184 692.96	58.8
	Frail	10	23	2.30	5794.20	5521.24	57 942.00	18.4
	Very frail	4	9	2.25	9272.75	8397.76	37 091.00	11.8
	**Total**	**40**	**100**	**2.50**	**7855.77**	**6822.48**	**314 230.92**	**100,0**
Total	Robust	47	55	1.17	3817.19	3198.23	179 407.93	20,6
	Pre-frail	62	96	1.55	7062.89	6707.16	437 899.18	50,2
	Frail	31	44	1.42	4643.9	3961.64	143 960.9	16,5
	Very frail	18	23	1.28	6140.28	4453.17	110 525.04	12,7
	**Total**	**158**	**218**	**1.38**	**5517.67**	**5263.30**	**871 792.12**	**100,0**

Some differences can be highlighted between the individuals who experienced a single or multiple HAs: the latter scored better than the former in all sections of the questionnaire (lower impairment in physical and mental health and better functional status and economic resources) except for social resources (S1 Fig and S3 Table). It is worth noting that those who experienced more than one HA showed an increased prevalence of diabetes compared to those who experienced only one HA (31.0% vs. 15.7%, p = 0.037) and also an increased prevalence of Parkinson’s Disease (7.1% vs 0; p = 0.005).

## Discussion

The main novelty of this study is the stratification of UHS cost by multidimensional bio-psychosocial level of frailty in a sample of older adults over one year of follow-up. Peters and colleagues underlined the association between frailty and increased health care costs (30). However, they did not consider the differences arising from the various levels of frailty. Other studies on the cost of hospitalization according to the level of frailty of older adults are based on a measurement of physical frailty which does not take into consideration the role of social and economic health determinants on the state of frailty [21–29].

The main determinant of the total UHS costs remains the number (or the rate per person/year) of HA [7–9]. It is noteworthy that pre-frail individuals account for the highest percentage of costs generated by UHSs, as well as for the highest number of used hospital services. This class of subjects did not show relevant differences in terms of psycho-physical health compared with the robust individuals at enrolment, except for the lack of social resources: for example 32.8% of them lived alone against 7.7% for the robust subjects (p<0.001) (S4 Table) (p<0.001). In fact, the analysis of determinants of HA rate carried out on the same sample showed a strong association between scarcity of social resources and a higher HA rate [19]. At the same time, the lack of association between diseases and expenditures underlines once more the need to overcome the concept of specific diseases as the main determinants of care costs. The lack of statistically significant association between frailty and increase of UHS cost is also consistent with these observations since the highest cumulative number of HAs in the observed sample is generated by sub-samples with an intermediate frailty score (frail and pre-frail individuals), resulting in the lack of a linear association (Pearson correlation = -0.04; p = 0.458).

Pre-frailty is considered a reversible condition [35] because it is usually associated with initial psycho-physical impairment or lack of socio-economic resources. Therefore, the implementation of preventative interventions targeting individual vulnerabilities is likely to have a significant impact on UHSs, as well as on their costs. In effect, even social interventions can reduce both the use and cost of hospital care by older adults [36,37].

Hospital care represents a major organizational burden for the health systems, thus determining a significant percentage of health care cost. Defining the determinants of hospital care costs represents a key point from which to reduce the costs and make the system sustainable in the medium-to-long term. Until now, most of the predictive models are based on specific diseases or on multimorbidity [38–42]. According to this approach, the more that older adults are affected by multimorbidity, the more they will use hospital resources. However, the approach based on diseases/multimorbidity takes into account only the psycho-physical dimension without considering either the impact of diseases/multimorbidity on the patients’ quality of life and functional capacity, or the influence of their socio-economic conditions [19, 43–47]. Frailty can represent the way to individuate older people with or without multimorbidity who are vulnerable to adverse health and social outcomes [43] and who may benefit from a tailored approach to care [43]. Moreover, in our analysis multimorbidity is associated with increased hospital care rate and cost in the univariate analysis, but it loses significance in the multivariate one, because of the competing impact of frailty, especially of the socio-economic areas and of functional impairment (S5 Table) [19]. Evidence reported in the literature demonstrates that the association between multimorbidity and mortality is lost when adjusted for functional impairment [19, 45, 48, 49], unlike frailty [19, 50]. Frailty seems to represent a functional and dynamic dimension for the assessment of the individuals’ need to use health services, as well as the risk of dying, while multimorbidity is a sum of pathologies not necessarily associated with disability and functional impairment which affect individuals’ quality of life and their consequent use of services that involves costs for the health system [51–53]. The assessment of frailty in older people could be a key method to identify those subjects for whom an intervention can be foreseen in order to reduce negative outcomes, such as multiple HA, independently from their multimorbidity.

However, it is still likely that multimorbidity affects the increase of hospitalization cost: in fact, the only disease that played a marginal role in this analysis was COPD, i.e. a chronic condition which could quickly and easily result in an unstable clinical situation. The main differences between COPD and non-COPD patients admitted to the hospital are the mean number of diseases (mean number of diseases 5 vs. 3; T-Test <0.001), which was higher for the admitted COPD patients. Again, it is not the disease but the concomitance of diseases and the clinical complications that seem indirectly related to the cost of hospital care. Therefore, the complexity of care due to different diseases (no matter what the disease is) and the clinical complications seem to be the main associations to be taken into account to explain why COPD is independently associated with the increased hospital care cost in this sample.

The measurement of multidimensional frailty according to the bio-psycho-social model permits a better understanding of the dynamics of hospital services costs. In fact, this method of screening allows us to identify a subpopulation of pre-frail individuals, representing only 1.41% of the full sample, characterized by a concentration of socio-economic impairment and a higher number of multiple hospitalizations so as to require about 19% of the total cost related to hospitalization in our sample. This is one of the reasons why researchers are addressing the screening of frailty in order to identify populations at higher risk of hospitalization in order to implement actions aimed at reducing both hospitalization and costs and preventing decline in quality of life [54, 55]. Overall, all the patients who underwent multiple hospital admissions generated higher costs. Therefore, when a patient is accepted for the second time in an acute hospital service during the same year, an individualized plan of care should be automatically implemented in order to manage these patients at their home as much as possible. In our analysis the role played by the length of stay is strictly linked to the Italian system of economic reimbursement due by the Region to the hospital for hospitalizations. Based on the DRG system, the economic reimbursement to the hospital is higher when the length of stay exceeds a pre-established threshold.

One of the main limitations of this study is to have taken into consideration only the costs of the health system, and not those incurred by patients, caregivers, or other sections. Considering a healthcare system such as the Italian one, where the hospital plays a prominent role in health care, it is likely that the costs incurred by the patients are relatively negligible for the purposes of this study. However, their estimation would be useful to determine the scale of elderly resources needed to manage the costs of interventions aimed at reducing or preventing frailty. Moreover, this study has been carried out in an environment characterized by a low provision of community care services, limiting the possibility to extend the results to other settings with a more developed community care approach.

Additionally, for very frail patients, whose number is smaller, and for whom it is more difficult to reduce the level of frailty, it would be necessary to intervene on the cost of services, with the aim of reducing the cost per person. Further studies on cost effectiveness are recommended to assess health and social interventions such as the development of social networks, the use of systems for monitoring the patients’ health and social status, or the increase in the offer of home care services [36,37]. These interventions may prove to be more efficacious both in terms of quality of service and cost-effectiveness or cost-benefit.

Finally, it is necessary to analyze how and how often transitions occur from one state to another, usually in a negative direction. In fact, a strategy that would reduce costs and improve the quality of life would be to prevent such transition by intervening on the most significant aspects of frailty.

## Conclusions

Multidimensional frailty is quantitatively and qualitatively a strong predictor of the use and cost of UHSs, even over a short period of observation. The assessment of multidimensional frailty of older adults permits better targeting of the important pre-frail population as the main category on which to concentrate the interventions on frailty prevention, this population being responsible for the highest number of HAs and the highest percentage of costs generated. The main feature of pre-frail individuals is not related only to their clinical pattern of disease or to their physical condition, but also to socio-economic conditions; therefore, economic sustainability of the welfare system could be supported by an ecological approach based on the integration of social and care services. Multidimensional frailty assessment provides crucial information for planning services to meet the care needs of older adults and improve both the effectiveness of care and the appropriateness of resource allocation. Further studies are needed to measure the impact of integrated services on the cost of older adults’ care. The systematic screening of frailty could be the key issue in improving the management of chronic diseases in community-dwelling older adults.

## Supporting information

S1 Fig Score of FGE questionnaire areas and number of hospital admissions. (DOCX)

S1 Minimum Dataset (XLSX)

S1 Table Comparison of total UHS cost and logarithm transformation of the same variable. (DOCX)

S2 Table Multivariable linear regression model. (DOCX)

S3 Table Mean score of FGE areas according to the number of hospital admission in the first year of follow up. (DOCX)

S4 Table Cohabitants according to frailty status at the enrollment. (DOCX)

S5 Table Relation between comorbidity and frailty score with HAs rate. (DOCX)
