# Supplementary material for: Cost of hospital care for the older adults according to their level of frailty. A cohort study in the Lazio region, Italy
Source: PLoS One. 2019 Jun 11;14(6):e0217829. doi: 10.1371/journal.pone.0217829 (PMC6559705; doi:10.1371/journal.pone.0217829)
Supplement: S1 Fig — (DOCX) [file pone.0217829.s001.docx]

Suppl fig 1. Mean score of FGE areas according to the number of hospital admission in the first year of follow up (sample: 152 subjects)
