# Supplementary material for: Cost of hospital care for the older adults according to their level of frailty. A cohort study in the Lazio region, Italy
Source: PLoS One. 2019 Jun 11;14(6):e0217829. doi: 10.1371/journal.pone.0217829 (PMC6559705; doi:10.1371/journal.pone.0217829)
Supplement: S1 Table — (DOCX) [file pone.0217829.s003.docx]

|  | | TOTAL UHS cost | LOG of TOTAL UHS cost |
| --- | --- | --- | --- |
| N | Valid | 386 | 380 |
|  | Missing | 0 | 6 |
| Mean | | 2856,9796 | 2,9083 |
| Mean Standard Error | | 237,50820 | ,03871 |
| Median | | 523,5000 | 2,7232 |
| Mode | | 104,70 | 2,02 |
| SD. | | 4666,29571 | ,75458 |
| Variance | | 21774315,667 | ,569 |
| Asimmetry | | 3,272 | ,202 |
| Asimmetry Standard Error | | ,124 | ,125 |
| Curtosis | | 15,235 | -1,426 |
| Curtois Standard Error | | ,248 | ,250 |
| Range | | 36519,40 | 2,54 |
| Minimum value | | ,00 | 2,02 |
| Maximum value | | 36519,40 | 4,56 |
| Percentiles | 25 | 104,7000 | 2,0199 |
|  | 50 | 523,5000 | 2,7232 |
|  | 75 | 3811,1750 | 3,5843 |
|  |  |  |  |

**Suppl. Table 1: comparison of Total UHS cost and logarithm transformation of the same variable**
