# Supplementary material for: Cost of hospital care for the older adults according to their level of frailty. A cohort study in the Lazio region, Italy
Source: PLoS One. 2019 Jun 11;14(6):e0217829. doi: 10.1371/journal.pone.0217829 (PMC6559705; doi:10.1371/journal.pone.0217829)
Supplement: S2 Table — (DOCX) [file pone.0217829.s004.docx]

| **Suppl Table 2. Multivariable Linear Regression Model;**  **outcome variable: Logarithm of total UHS cost** | | | | | |  |
| --- | --- | --- | --- | --- | --- | --- |
|  | | | | | | |
| Model | R |  |  |  |  |  |
|  |  | Modifica R-quadrato | | Modifica F | Sign. Modifica F |  |
| Hospitalization Admission rate per person/year | 0.540 | 0.292 | | 151.264 | <0.001 |  |
| Length of stay (cumulative per person) | 0.553 | 0.014 | | 7.322 | 0.007 |  |
| Cancer | 0.560 | 0.008 | | 4.388 | 0.037 |  |
