# Supplementary material for: Cost of hospital care for the older adults according to their level of frailty. A cohort study in the Lazio region, Italy
Source: PLoS One. 2019 Jun 11;14(6):e0217829. doi: 10.1371/journal.pone.0217829 (PMC6559705; doi:10.1371/journal.pone.0217829)
Supplement: S3 Table — (DOCX) [file pone.0217829.s005.docx]

| Number of HAs | | Physical Health | Mental Health | Functional Status | Social Resources | Economic Resources |
| --- | --- | --- | --- | --- | --- | --- |
| 1 | Mean | -9.99 | -5.61 | 31.95 | 21.37 | 9.64 |
|  | N. | 109 | 109 | 109 | 109 | 109 |
|  | SD | 12.24 | 13.14 | 13.12 | 5.71 | 5.63 |
| >1 | Mean | -9.02 | -3.60 | 33.67 | 19.56 | 10.67 |
|  | N. | 43 | 43 | 43 | 43 | 43 |
|  | SD | 10.75 | 9.31 | 11.62 | 6.59 | 4.88 |
| Total | Mean | -9.72 | -5.04 | 32.44 | 20.86 | 9.93 |
|  | N. | 152 | 152 | 152 | 152 | 152 |
|  | SD | 11.81 | 12.19 | 12.70 | 6.00 | 5.43 |

Suppl table 3. Mean score of FGE areas according to the number of hospital admission in the first year of follow up (sample: 152 subjects)
