# Supplementary material for: Cost of hospital care for the older adults according to their level of frailty. A cohort study in the Lazio region, Italy
Source: PLoS One. 2019 Jun 11;14(6):e0217829. doi: 10.1371/journal.pone.0217829 (PMC6559705; doi:10.1371/journal.pone.0217829)
Supplement: S4 Table — (DOCX) [file pone.0217829.s006.docx]

|  | Cohabitants | | | | | Total |
| --- | --- | --- | --- | --- | --- | --- |
|  | alone | spouse | child | others | paid assistant |  |
| Very frail | 15 | 21 | 32 | 9 | 20 | 97 |
|  | 15.5% | 21.6% | 33.0% | 9.3% | 20.6% | 100.0% |
| Frail | 57 | 49 | 54 | 8 | 7 | 175 |
|  | 32.6% | 28.0% | 30.9% | 4.6% | 4.0% | 100.0% |
| Pre-frail | 151 | 196 | 96 | 12 | 5 | 460 |
|  | 32.8% | 42.6% | 20.9% | 2.6% | 1.1% | 100.0% |
| Robust | 42 | 398 | 93 | 14 | 1 | 548 |
|  | 7.7% | 72.6% | 17.0% | 2.6% | .2% | 100.0% |
| Total | 265 | 664 | 275 | 43 | 33 | 1280 |
|  | 20.7% | 51.9% | 21.5% | 3.4% | 2.6% | 100.0% |

Suppl Tab 4. Cohabitants according to frailty status at the enrollment
