# Supplementary material for: Cost of hospital care for the older adults according to their level of frailty. A cohort study in the Lazio region, Italy
Source: PLoS One. 2019 Jun 11;14(6):e0217829. doi: 10.1371/journal.pone.0217829 (PMC6559705; doi:10.1371/journal.pone.0217829)
Supplement: S5 Table — (DOCX) [file pone.0217829.s007.docx]

|  | Univariable | Multivariable |
| --- | --- | --- |
|  | Pearson corr. | ß |
| Comorbidity | 0,052 p=0.031 | 0.019  p=0.528 |
| Frailty score | -0,112 p<0.001 | -0.106 p<0.001 |

Suppl Table 5. Relation between comorbidity and frailty score with HAs rate
